# Antimicrobial Properties of Analogs of Dimeric A-Type Proanthocyanidins in Food Matrices

**DOI:** 10.3390/ijms27020853

**Published:** 2026-01-15

**Authors:** Antonio Cobo, Alfonso Alejo-Armijo, Daniel Cruz, Cristina Cuadrado, Elena Ortega-Morente

**Affiliations:** 1 Department of Health Sciences, Faculty of Experimental Sciences, University of Jaén, Campus of International Excellence in Agri-Food (ceiA3), 23071 Jaén, Spain; acmolinos@ugr.es (A.C.); dcsaez@ujaen.es (D.C.); 2 Department of Inorganic and Organic Chemistry, Faculty of Experimental Sciences, University of Jaén, Campus of International Excellence in Agri-Food (ceiA3), 23071 Jaén, Spain; aalejo@ujaen.es (A.A.-A.); ccg00051@red.ujaen.es (C.C.)

**Keywords:** A-type proanthocyanidins, antimicrobial activity, synergistic activities, food matrices, food preservatives

## Abstract

Polyphenols with antimicrobial and antibiofilm properties are gaining popularity due to their natural origins and relatively safe nature, and they have met the interest of the food industry because of their possible applicability as food preservatives. We have investigated the effect of different analogs of dimeric A-type proanthocyanidins (PACs) on four food matrix models, including unprocessed meat, fish, vegetables and dairy products previously contaminated with susceptible food pathogens. The best effects were achieved when cherry tomato was used as the food matrix for all the target bacteria (*Staphylococcus aureus* CECT 828, *Listeria innocua* CECT 910 and *Bacillus cereus* UJA27q) and for both temperatures tested (6 and 25 °C). Moreover, several combinations of these analogs also showed synergistic effects, mainly on *S. aureus* CECT 828, which may allow these antimicrobials to be used at lower levels in food matrices, which would promote their sensory acceptability. However, further studies should be conducted next to understand the mechanisms of these synergistic activities between the phenolic compounds against foodborne pathogens, as well as to ensure the absence of toxic effects when used as food preservatives.

## 1. Introduction

Novel natural antimicrobial agents have been thoroughly investigated in recent decades because of the increase in multidrug-resistant microbes and the growing failure of conventional pharmaceutical antibiotics in clinical practice [1]. Polyphenols with antimicrobial and antibiofilm properties are gaining popularity due to their natural origins and relatively safe nature [2], as phenolic compounds are considered the largest group of plant secondary metabolites and some of the most interesting bioactive compounds. They have met the interest of the food industry not only because of their antioxidant capacity [3] but also owing to their antimicrobial activity [4,5], especially in seafoods [6]. As for the mechanism of action, previous studies suggest that it may be mediated by changing intracellular functions, modifying cell membranes’ permeability or inducing the loss of the cell wall integrity [7].

In that sense, some analogs to dimeric A-type proanthocyanidins (PACs), a class of natural polyphenols, have shown promising antimicrobial and antibiofilm activities against foodborne bacteria [8,9]. Previous studies also suggested that chitosan films added to PACs exhibit improved antioxidant and antimicrobial activity in comparison with plain chitosan films, so these compounds could also be applied as active packaging materials in the food industry [10].

However, discrepancies in the effectiveness of these compounds against microorganisms in laboratory conditions and in food systems is a major concern in their utility, as substances with high antimicrobial activity in vitro may have little or no effect in food matrices due to their interaction with food components [11,12]. Moreover, the difference in susceptibility to polyphenols between Gram-positive and Gram-negative bacteria is a controversial issue, as polyphenols are generally more effective against Gram-positive bacteria, probably due to the outer complex membrane of Gram-negative bacteria, which slows down the passage of chemicals, although this outer membrane may also be altered by some polyphenols [13].

**Figure 1:**
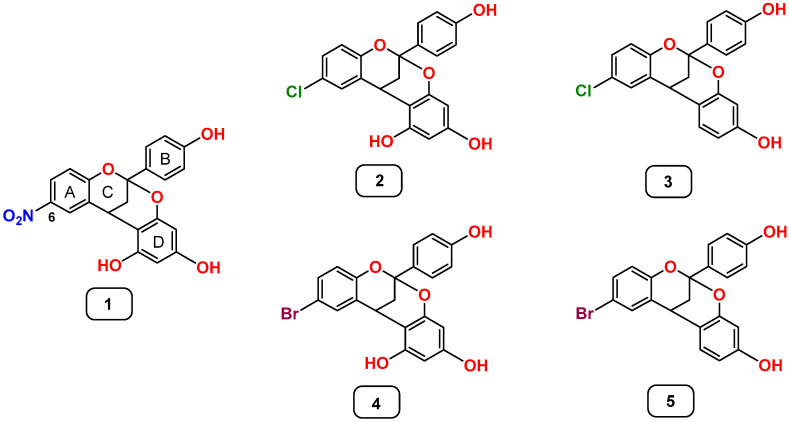
Analogs to dimeric A-type proanthocyanidins (PACs) evaluated in this work on food matrix models.

Based on our previous results on the antimicrobial efficacy of different analogs on PACs on foodborne pathogens, we have investigated the effect of the most active compounds on four food matrix models, including unprocessed meat, fish, vegetables, and dairy products previously contaminated with susceptible food pathogens. The selected target strains were Gram-positive bacteria because of their importance as food pathogens as well as for their being the most susceptible to the compounds tested. Of all the compounds investigated in vitro by us in the past [8,9], we have selected the five most active ones for this work, which are gathered in Figure 1.

Compound **1** contains a nitro group (NO_2_) at carbon 6 of ring A and three hydroxyl groups (OH) distributed in rings B and D. Compound **2** is identical to **1** in the number and distribution of OHs, but contains a chlorine atom (Cl) at C-6 instead of an NO_2_ group. Compound **3** is like **2** but with one less OH group at ring D. Compounds **4** and **5** are identical to **2** and **3**, respectively, but with a bromine atom (Br) instead of a chlorine at C-6.

With the purpose of obtaining analogs with high antibacterial activity and studying the influence of the substitution pattern on rings B–D, we had previously envisioned seven analogs to A-type PACs, all of them with a nitro group at A ring and with one or two hydroxyl groups on rings B and D, and with a methyl group at C-ring or without [8]. Next, we reported the synthesis of eight additional analogs with chloro and bromo atoms at the A-ring and described the systematic study of their antimicrobial and antioxidant activities in order to evaluate their possible application as biocides or food preservatives, as well as to elucidate new structure–activity relationships [9]. Among those analogs, the five selected for this assay had showed the highest antibacterial effect when assayed in vitro, showing MICs of 10 μg/mL against the target strains, excluding compound **1** against *Staphylococcus aureus* CECT 828 and *Bacillus cereus* UJA27q, Compound **2** against *B. cereus* UJA27q and Compound **3** against *S. aureus* CECT 828, all of which showed MICs of 50 μg/mL against the target bacteria.

We report herein the effect of those five analogs in food matrices. The best effects are achieved when cherry tomato is used as food matrix for all the target bacteria and at both tested temperatures. Compounds **1** and **2** stand out because of their effect on *S. aureus* CECT 828, while Compounds **3** and **4** show the best effect on *Listeria innocua* CECT 910 and Compounds **4** and **5** obtained the highest growth inhibition on *B. cereus* UJA27q. Moreover, several combinations showed synergistic effects mainly on *S. aureus* CECT 828, although some combinations also showed high antimicrobial activity against *B. cereus* UJA27q and *L. innocua* CECT 910.

## 2. Results

### 2.1. Antibacterial Effect of Compounds on Food Matrices

In this paper, we have used different food models in order to investigate the antibacterial activity of analogs to previously synthesized and analyzed PACs in vitro in terms of their antibacterial and antibiofilm activity against foodborne pathogens [8,9,14].

**Figure 2:**
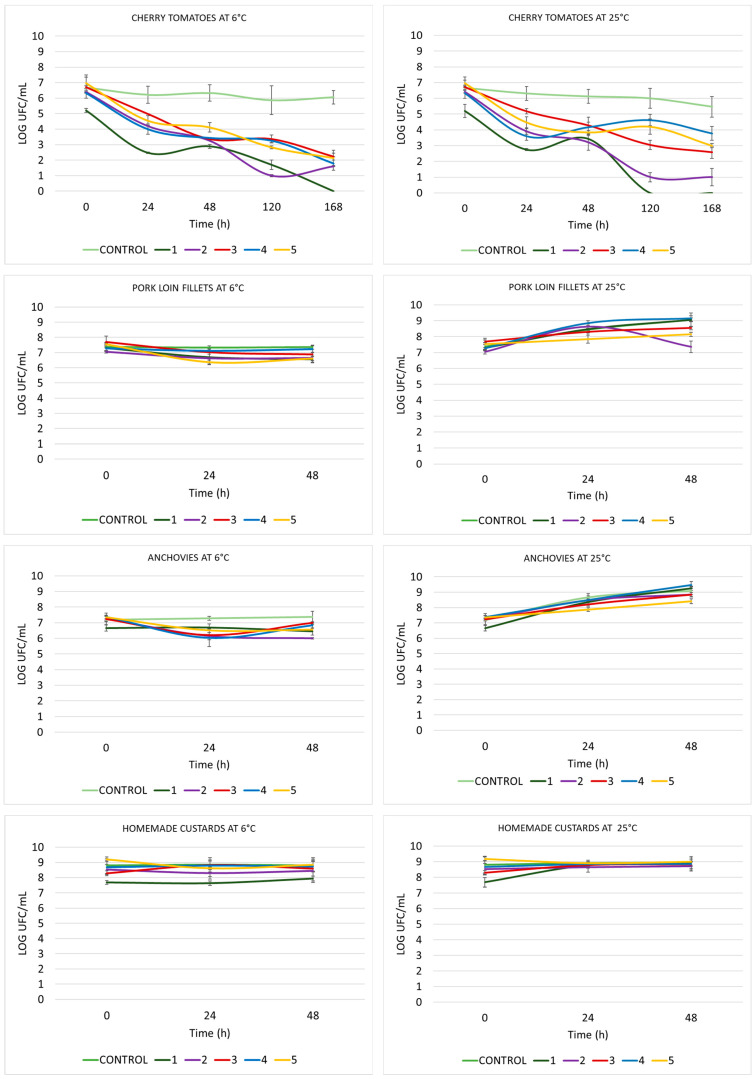
The counts of viable *Staphylococcus aureus* CECT 828.

As shown in Figure 2, the counts of viable *S. aureus* CECT 828 in cherry tomato were between 6 and 7 log throughout the experiment in control samples. When the samples were treated with compound **1**, viable cell counts significantly decreased to 2.4 log at 24 h and to undetectable levels after 120 h at 25 °C and after 168 h at 6 °C. Compound **2** was able to decrease bacterial counts to just one log at 120 h at both tested temperatures, although it could not reach undetectable levels at 168 h. Treatment with compound **3** decreased bacterial counts for 4 logs at the end of the experiment and this decrease was gradual over time at 25 °C and especially significant after 48 h of incubation at 6 °C. Compound **4** induced significant decreases in bacterial counts at 24 and 168 h at both tested temperatures. Finally, compound **5** decreased bacterial counts especially at 24 h, with final counts of 2 logs at 6 °C and 3 logs at 25 °C at the end of the experiments. In general terms, the effects of the treatments were more significant at 6 °C and compound **1** stands out for being able to decrease bacterial counts to undetectable levels at both tested temperatures.

Regarding other assayed matrices contaminated with *S. aureus* CECT 828, both the control and treated samples showed similar viable cell counts throughout the entire experiment, with no significant effects for any of the compounds tested.

**Figure 3:**
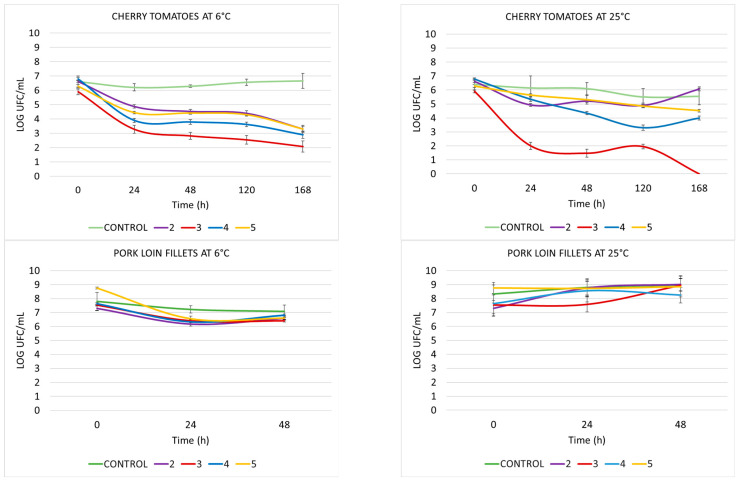

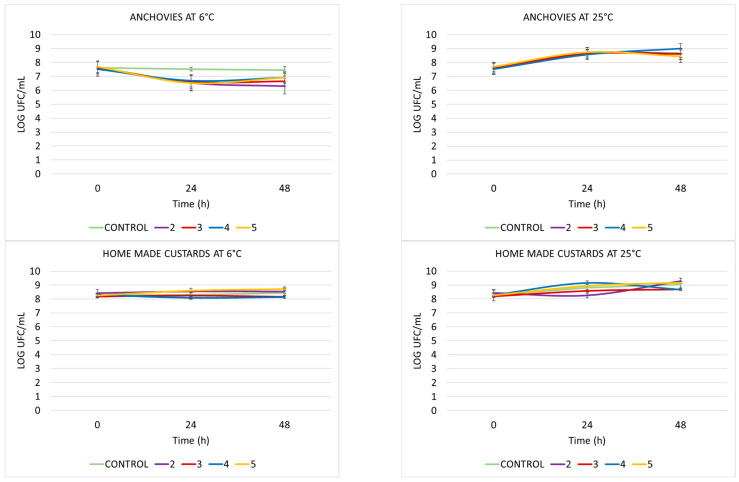
The counts of viable *Listeria innocua* CECT 910.

Viable counts of *L. innocua* CECT 910 in the different matrices assayed are shown in Figure 3. When cherry tomato was used as a food matrix, control samples showed viable counts of approximately 6 logs throughout the entire experiment. Treatment with all compounds tested at 6 °C induced a decrease in viable cell counts of 2 to 3 logs at 24 and 168 h, with a total decrease between 3 and 4 logs at the end of the experiment. When samples were incubated at 25 °C, compound **3** stood out with a 4-log decrease in bacterial count at 24 h and reached undetectable levels after 168 h of incubation. Compound **4** induced a gradual reduction on cell counts, with a maximum effect (of 3-log reduction) at 120 h of incubation at 25 °C.

Control and treated samples from the other food matrices assayed showed similar viable cell counts throughout the experiment, excluding cellular counts from pork loin filets and anchovies at 6 °C, which showed a decrease of 2 logs at 24 h for all the compounds tested. However, viable cell counts recovered at 48 h and no significant differences were achieved for any of the applied treatments at the end of the experiments.

**Figure 4:**
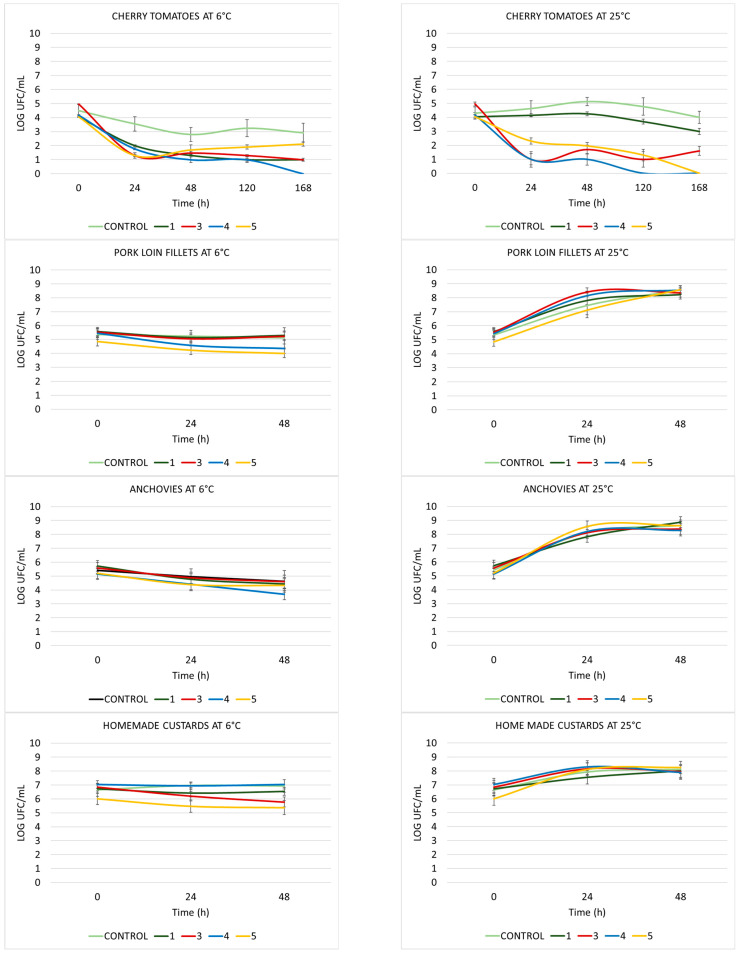
The counts of viable *Bacillus cereus* UJA27q.

Viable cell counts of *B. cereus* UJA27q are shown in Figure 4. When pork loin, anchovies or homemade custards were used as food matrices, similar counts were observed in control and treated samples, both at 24 and 48 h, with no significant reductions after treatments.

On the contrary, viable cell counts of cherry tomatoes incubated at 6 °C showed significant reductions (of 3 to 4 logs) 24 h after treatment with all the compound tested (**1**, **3**–**5**). Treatment with compound **4** was also able to decrease viable cell counts to undetectable levels after 168 h of incubation; the same was observed with compounds **4** and **5** when the samples were incubated at 25 °C. Compound **3** showed a similar effect at both temperatures tested and compound **5** showed a gradual reduction in viable cell counts with undetectable levels at the end of the assay at 25 °C. Finally, samples treated with compound **1** showed similar viable cell counts to control samples, with just one log of reduction throughout the experiment.

Briefly, the best effects are achieved when cherry tomato is used as the food matrix for all the target bacteria and at both tested temperatures. Compounds **1** and **2** stand out because of their effect on *S. aureus* CECT 828, while compounds **3** and **4** show the best effect on *L. innocua* CECT 910 and compounds **4** and **5** obtained the highest growth inhibition on *B. cereus* UJA27q.

### 2.2. Synergistic Effects

Our results of the checkerboard method are shown in Table 1, Table 2 and Table 3. Synergistic effects were found for compound **1** with all the other tested compounds on *S. aureus* CECT 828, as well as for combinations of compounds **2** and **3** and compounds **3** and **4**, as well as for compounds **4** and **5**. All these couples of compounds showed isoeffective combinations with similar effects to the compounds when acting alone (Table 1).

When *B. cereus* UJA27q was used as target strain (Table 2), synergistic effects were found between compound **1** and compounds **3**–**5**, as well as between compound **3** and compounds **4** and **5**.

Finally, compound **2** combined with compounds **3** and **5**, as well as compound **3** in association with compound **5,** also showed synergistic effects against *L. innocua* CECT 910 (Table 3).

## 3. Discussion

Antimicrobial and antioxidant packaging play an important role in the food industry by prolonging the shelf life of products as well as ensuring food quality. The development of active food packaging using natural products with antimicrobial and antioxidant properties and the advantages of incorporating natural products into polymer matrices to develop industrial packaging has induced technological advances in this field of knowledge and its applicability in the food industry [15]. In this field of research, the antimicrobial activity of phenolic compounds is part of the scientific discussion regarding the use of natural plant extracts as alternative food preservative agents, as phenolic compounds that can be derived from a range of fruits and vegetables, including tea, coffee, chocolate and wine are effective natural additives that delay oxidative processes and the growth of microorganisms [16]. Consequently, they facilitate the development of fresh, healthy, and safe food products with a prolonged shelf life, although there is still little information regarding their applicability for food preservation. This might be primarily due to the lack of information regarding the full antimicrobial spectrum of the compounds, their synergisms in natural or artificial combinations, and their interaction with food ingredients [17]. In this sense, the type of food matrix definitely influences the effectiveness of phenolic compounds as food preservatives. It has been previously described that polyethylene films coated with propolis ethanolic extract exhibit different antimicrobial effects on *Listeria monocytogenes*-contaminated cheese, salmon, and salami slices, indicating that the antimicrobial activity of the films is influenced by the type of food [18]. Studies on different food models focused on the interaction of the phenolic compounds with the protein in the products are especially necessary, as previous studies [19,20,21,22] have found that nitrogenous compounds reduce the antimicrobial activity of phenolic compounds contained in natural extracts and its derivatives. High-protein food matrices may attenuate the biological activity of phenolic compounds due to the formation of stable polyphenol–protein complexes, which reduce the free fraction, bioaccessibility, and effective interaction of phenolics with their biological targets [23]. Accordingly, the preserving effects of these compounds might be diminished in foods with high protein contents, which support the limited results found in the present study when meat, fish and dairy matrices were evaluated, compared to vegetal ones. Moreover, the effectiveness of these compounds when directly added to food may decrease due to several factors, such as poor solubility, inactivation by pH or oxidation processes, the adsorption of the compound to certain components, degradation by enzymes or irregular distribution in the food matrix, among others [24,25,26]. On the other hand, a detailed study of the interactions between proteins (P) and phenolic acids (PA) can lead to the creation of new food formulations with new properties, as the oxidative and thermal stability of the protein–phenolic acid (PPA) conjugates are improved in these systems, and there are also reports of antimicrobial properties of PPA conjugates [27]. Moreover, thermal and non-thermal treatments may also induce alterations in those interactions [28]. Therefore, further specific research should be conducted on different types of food matrices and using a wide range of products to ensure the effectiveness of phenolic compounds as food preservatives.

In this study, the best results were found when cherry tomatoes were used as the food model, while matrices with higher protein contents showed limited results. Gram-positive bacteria had been previously described to be the most sensitive to all the analogs and combinations assayed, showing MICs from 10 to 50 µg/mL, so three Gram-positive strains were used in our assays. When applied on food matrices previously contaminated with *S. aureus* CECT 828, the best results were found at 6 °C, and compounds **1** and **2** stand out as the most effective. These results in food models support those previously described in vitro [8,9], so the analogs with only one OH group at B-ring and with three OH groups in total are more active than those with four OH groups, not only in the nitro derivative (compound **1**) but also in the halogenated analogs to natural A-type PACs (compounds **2** to **5**). When *L. innocua* CECT 910 was used as the target bacteria, compounds **3** and **4** were the most active and samples incubated at 25 °C showed undetectable levels of bacterial growth after 168 h of incubation with compound **3**. In this instance, halogenated analogs to natural A-type PACs rose above the nitro derivatives, as previously reported when in vitro assays were performed to evaluate their antimicrobial activities against these bacteria. Finally, viable cell counts of *B. cereus* UJA27q in cherry tomatoes were considerably reduced by compounds **3**–**5** at both temperatures tested, while compound **1** showed similar viable cell counts to the control sample at 25 °C. Again, those analogs to natural A-type PACs with chloro or bromo atoms at the A-ring show better activity than the nitro derivative.

Several studies have also been conducted to investigate the possible synergistic effect of different natural compounds in combination with each other, as well as with other antimicrobials, with the aim of reducing their concentration in food products and increasing their antimicrobial efficacy in food matrices [29,30,31]. Phenolic compounds, when used in elevated concentrations in food products, are of limited use due to an important hurdle in sensory acceptance [32], so synergistic antimicrobial effects have been investigated from both polyphenols and polysaccharides of films for food packaging [33], e.g., chitosan-based films fortified with the polyphenols from young apple show better antimicrobial activities [34]. The synergistic effects observed may be explained by a combination of chemical mechanisms. In that sense, the coexistence of structurally related phenolic units enables multivalent interactions with biological targets through hydrogen bonding, hydrophobic interactions, and π–π stacking, thereby increasing the apparent binding affinity compared to individual compounds. In addition, complementary redox properties among phenolic moieties may allow regeneration of the most active species, prolonging their functional activity. Together, these combined chemical and biological interactions provide a plausible basis for the synergistic effects observed [35].

The structure–function relationships of many polyphenols and polysaccharides have been studied in detail and should be of great interest to guide the selection of appropriate combinations for the antioxidant and antimicrobial applications of films and coatings. These synergistic combinations can also allow these antimicrobials to be used at a lower level in food matrices that would meet sensory acceptability. Herein, we describe synergistic effects among several compounds on all the bacterial target studied, with lower isoeffective combinations capable of inhibiting the growth of microorganisms at the same level as compounds when acting alone. Nonetheless, further studies should be conducted to better understand the mechanisms of these synergistic activities against foodborne pathogens between not only the phenolic compounds and some food components but also with polysaccharides from food packaging, to estimate their affinity and stability while exhibiting suitable formulations that can lead to increased antimicrobial activities and extended shelf life of foods. Finally, the impact of food processing on the stability and bioavailability of the phenolic compounds also needs to be considered, which is crucial to ensuring that the antimicrobial properties of these compounds are maintained throughout food packaging and storage. Optimized processing techniques and innovative technologies, such as fermentation, controlled thermal processing or nanoencapsulation should be used in order to minimize degradation while enhancing the bioavailability and functionality of these compounds.

## 4. Materials and Methods

### 4.1. Target Strains

Target strains were selected because of their importance as food pathogens as well as for being the most susceptible to the compounds analyzed, based on our previous results.

They include strains from the Spanish Type-Culture Collection (CECT) (*S.aureus* CECT 828 and *L. innocua* CECT 910), as well as a multidrug-resistant strain of our own collection from organic foods (*B. cereus* UJA27q).

### 4.2. Analogs Tested

The compounds tested in the present study (Figure 1) were previously synthesized and characterized by the authors [8,9,14]. They were selected based on their high antimicrobial activity detected in previous assays, which are summarized in Table 4.

### 4.3. Antimicrobial Activity on Food Matrix

Four food matrix models, including unprocessed meat, fish, vegetables and dairy products previously contaminated with susceptible food pathogens, have been used in the experiments. Specifically, pork loin filets, anchovies, organic cherry tomatoes and homemade custards were used as food matrices in order to detect the possible variability of the effects of these analogs depending on the type and composition of the foods susceptible to contamination with the bacterial targets that we have analyzed.

#### 4.3.1. Preparation of Analog Solutions

Immersion solutions containing 10 or 50 µg/mL of each testing analog in distilled water were obtained depending on the Minimal Inhibitory Concentration (MIC) values previously obtained for each target strain. All solutions were prepared fresh and kept at room temperature for at least 10 min before use.

#### 4.3.2. Determination of the Effectiveness of Analogs on Contaminated Food Matrix Models

The effect of immersion in solutions containing MICs of the analogs on survival and growth of strains inoculated onto pork loin filets, anchovies and cherry tomatoes from local supermarkets, as well as onto homemade custards, was investigated at two storage temperatures (6 and 25 °C). Contaminating solution was obtained by dilution to 1:10 in sterile saline solution with the cultures of the target strains grown overnight in Trypto-Casein Soy Broth (TSB; Panreac, Barcelona, Spain) at 37 °C, in order to reach a final cell density of 7 log CFU/mL.

For treatment application, pork loin filets and anchovies were cut onto 2 × 2 cm pieces. Full pieces of cherry tomatoes and 5 mL of homemade custards were used for the experiments. All samples were deposited inside sterile capped 50 mL polypropylene tubes (Sterilin, Stone, GB) and dipped for 5 min in 5 mL of each strain-contaminated solution at room temperature. Samples were then drained for removal of the excess contaminating solution and the contaminated samples were next immersed for 5 min at room temperature in 5 mL of sterile distilled water (controls) or in 5 mL of solutions containing the corresponding MICs of analogs. After immersion treatments, the samples were drained in order to remove the excess treatment solution and the samples were stored in 50 mL polypropylene test tubes with sterile caps in refrigerated storage (at 6 °C) or in incubation chambers (at 25 °C) after sampling to obtain viable cell counts of the target strains at the initial time in each tested matrix.

Cell counts were also performed at different sampling times (24 and 48 h for meat, fish and dairy samples and 24, 48, 120 and 168 h for tomato samples). Treated samples, stored at different temperatures, were mixed with 5 mL of sterile saline (0.85% NaCl) and processed for 3 min in a Stomacher 80 (Biomaster, Madrid, Spain). They were then serially diluted in sterile saline and spread in triplicate onto selective agar plates for each bacterial species tested: Vogel Johnson Agar supplemented with sodium tellurite (Scharlab, Barcelona, Spain) for *S. aureus* CECT 828, PALCAM Agar added with *Listeria* supplement (Panreac) for *Listeria innocua* CECT 910, and *B. cereus* agar (Scharlab) supplemented with egg yolk and polymyxin B (Panreac) for *B. cereus* UJA27q. The plates were incubated at 37 °C for 48 h, and viable cell counts were determined.

### 4.4. Checkerboard Titer Tests

The checkerboard method was used in order to search for possible synergistic effects between the most active analogs against food pathogens in the corresponding target matrix. Two-fold sequential dilutions of the active compounds grouped in pairs were tested against the target strains, according to the methodology previously described for individual compounds. Isoeffective combinations were considered to be those that showed similar log reductions in bacterial counts to the analogs when acting alone, and for all counting times, from 24 to 168 h.

The results are expressed as the sum of the fractional inhibitory concentration (FIC) index for each agent (fractional number resulting from the active concentration of an agent in combination divided by the active concentration of this compound alone). The FIC value of the most effective combination is used in calculating the fractional inhibitory concentration index (FICI) by adding both FICs: FICI = FICA + FICB = CAcomb/CAalone + CBcomb/CBalone, where CAalone and CBalone are the active concentrations of compounds A and B when acting alone and CAcomb and CBcomb are concentrations of compounds A and B at the isoeffective combinations, respectively. The FICI was interpreted as synergistic activity when it was ≤0.5, antagonistic when it was >4.0, and any value in between was interpreted as indifferent [36,37].

### 4.5. Statistical Analysis

The average data and standard deviations from counts of viable cells were determined with the Excel program version 18.0 (Microsoft Corp., Redmond, WA, USA). A t-test was performed at the 95% confidence level with Statgraphics Plus version 5.1 (Statistical Graphics Corp., Rockville, MD, USA) to determine the statistical significance of the data.

## 5. Conclusions

This study analyzes the effectiveness of analogs to dimeric A-type proanthocyanidins and their combinations in food matrices, showing the best results in cherry tomatoes and supporting the development of novel food preservative agents derived from natural substances, once the cytotoxic effects have been discarded by undertaking toxic effect studies in cells and animals, as well as studies on coating and release profiles. Thus, phenolic compounds may offer a promising opportunity in the field of innovative food preservation as a means of satisfying consumer demand for nutritious and fresh food products.

## Figures and Tables

**Table 1:** Checkerboard assay of analogs **1**–**5** against *Staphylococcus aureus* CECT 828 in cherry tomatoes.

MIC of Each Agent (μg/mL)					
Compound	Alone	Combination	FIC	FICI	Outcome
**1**	50	12.5	0.25		
**2**	10	1.25	0.125	0.375	SYN
**1**	50	6.25	0.125		
**3**	10	1.25	0.125	0.25	SYN
**1**	50	6.25	0.125		
**4**	10	1.25	0.125	0.25	SYN
**1**	50	12.5	0.25		
**5**	10	2.5	0.25	0.5	SYN
**2**	10	2.5	0.25		
**3**	10	1.25	0.125	0.375	SYN
**2**	10	2.5	0.25		
**4**	10	2.5	0.25	0.5	IND
**2**	10	2.5	0.25		
**5**	10	2.5	0.25	0.5	IND
**3**	10	2.5	0.25		
**4**	10	1.25	0.125	0.375	SYN
**3**	10	2.5	0.25		
**5**	10	2.5	0.25	0.5	IND
**4**	10	2.5	0.25		
**5**	10	1.25	0.125	0.375	SYN

MIC: minimal inhibitory concentration; FIC: fractional inhibitory concentration (FIC = MIC combination/MIC alone); FICI = sum of the FIC index for each compound. SYN: synergy. IND: indifferent.

**Table 2:** Checkerboard assay of analogs **1**–**5** against *Bacillus cereus* UJA27q in cherry tomatoes.

MIC of Each Agent (μg/mL)					
Compound	Alone	Combination	FIC	FICI	Outcome
**1**	50	6.25	0.125		
**3**	10	1.25	0.125	0.25	SYN
**1**	50	6.25	0.125		
**4**	10	2.5	0.25	0.375	SYN
**1**	50	6.25	0.125		
**5**	10	2.5	0.25	0.375	SYN
**3**	10	1.25	0.125		
**4**	10	2.5	0.25	0.375	SYN
**3**	10	2.5	0.25		
**5**	10	1.25	0.125	0.375	SYN
**4**	10	2.5	0.25		
**5**	10	2.5	0.25	0.5	IND

MIC: minimal inhibitory concentration; FIC: fractional inhibitory concentration (FIC = MIC combination/MIC alone); FICI = sum of the FIC index for each compound. SYN: synergy. IND: indifferent.

**Table 3:** Checkerboard assay of analogs **1**–**5** against *Listeria innocua* CECT 910 in cherry tomatoes.

MIC of Each Agent (μg/mL)					
Compound	Alone	Combination	FIC	FICI	Outcome
**2**	10	2.5	0.25		
**3**	10	1.25	0.125	0.375	SYN
**2**	10	2.5	0.25		
**4**	10	2.5	0.25	0.5	IND
**2**	10	1.25	0.125		
**5**	10	2.5	0.25	0.375	SYN
**3**	10	2.5	0.25		
**4**	10	2.5	0.25	0.5	IND
**3**	10	1.25	0.125		
**5**	10	1.25	0.125	0.25	SYN
**4**	10	2.5	0.25		
**5**	10	2.5	0.25	0.5	IND

MIC: minimal inhibitory concentration; FIC: fractional inhibitory concentration (FIC = MIC combination/MIC alone); FICI = sum of the FIC index for each compound. SYN: synergy. IND: indifferent.

**Table 4:** MICs of analogs against target strains according to previous studies (µg/mL).

Compound	*S. aureus* CECT 828	*L. innocua* CECT 910	*B. cereus* UJA27q
**1**	50	^a^	50
**2**	10	50	^a^
**3**	10	10	50
**4**	10	10	10
**5**	10	10	10

^a^ MIC was above 1 mg/mL.

## Data Availability

The original contributions presented in this study are included in the article. Further inquiries can be directed to the corresponding authors.
